# Fostering citizen‐engaged HIV implementation science

**DOI:** 10.1002/jia2.26278

**Published:** 2024-07-05

**Authors:** Benedict Xin Hao Tan, Shao Yuan Chong, Daniel Weng Siong Ho, Ye Xuan Wee, Muhammad Hafiz Jamal, Rayner Kay Jin Tan

**Affiliations:** ^1^ Rainbow+ Singapore Singapore; ^2^ Independent Scholar Singapore Singapore; ^3^ Saw Swee Hock School of Public Health National University of Singapore and National University Health System Singapore Singapore

**Keywords:** citizen science, community engagement, stakeholder engagement, participatory research, qualitative research, health systems

## Abstract

**Introduction:**

Successful implementation of evidence‐based practices depends on contextual factors like stakeholder engagement, the socio‐political environment, resource availability, and stakeholders’ felt needs and preferences. Nevertheless, inequities in implementation exist and undermine efforts to address HIV in marginalized key populations. Implementation science shows promise in addressing such inequities in the HIV response, but can be limited without meaningful engagement from citizens or communities.

**Discussion:**

We define the concept of a citizen‐engaged HIV implementation science as one that involves citizens and communities deeply in HIV implementation science activities. In this commentary, we discuss how citizen science approaches can be leveraged to spur equity in HIV implementation science. Drawing on three areas previously defined by Geng and colleagues that serve to drive impactful implementation science in the HIV response, we discuss how citizens can be engaged when considering “whose perspectives?”, “what questions are being asked?” and “how are questions asked?”. With respect to “whose perspectives?” a citizen‐engaged HIV implementation science would leverage participatory methods and tools, such as co‐creation, co‐production and crowdsourcing approaches, to engage the public in identifying challenges, solve health problems and implement solutions. In terms of “what questions are being asked?”, we discuss how efforts are being made to synthesize citizen or community‐led approaches with existing implementation science frameworks and approaches. This also means that we ensure communities have a say in interrogating and deconstructing such frameworks and adapting them to local contexts through participatory approaches. Finally, when considering “how are questions asked?”, we argue for the development and adoption of broad, guiding principles and frameworks that account for dynamic contexts to promote citizen‐engaged research in HIV implementation science. This also means avoiding narrow definitions that limit the creativity, innovation and ground‐up wisdom of local citizens.

**Conclusions:**

By involving communities and citizens in the development and growth of HIV implementation science, we can ensure that our implementation approaches remain equitable and committed to bridging divides and ending AIDS as a public health threat. Ultimately, efforts should be made to foster a citizen‐ and community‐engaged HIV implementation science to spur equity in our global HIV response.

## 1 INTRODUCTION

Successful implementation of evidence‐based practices depends on contextual factors like stakeholder engagement, the socio‐political environment, resource availability, and stakeholders’ felt needs and preferences [1, 2]. Implementation science has been defined as “the scientific study of methods to promote the systematic uptake of research findings and other evidence‐based practices into routine practice, and, hence, to improve the quality and effectiveness of health services” [3]. When applied to HIV science, implementation science examines the factors that impact the uptake, diffusion, scaling up, sustainability and effectiveness of HIV programmes and services [4, 5]. This examination is particularly crucial among marginalized and key populations inequitably affected by HIV [6, 7, 8]. These inequalities suggest that despite successes in developing intervention strategies, the barriers to implementing these strategies outside the research setting must be examined and addressed for population‐level impact to be observed [9, 10].

Implementation science can address these inequalities, but several factors may limit how we can bridge the gap between lab‐based research and real‐world practice. First, there are concerns about the extent to which implementation studies can account for the complexity of real‐world contexts or the resources and time required to support this research [11]. Second, structural factors may lead us to overlook research among the most marginalized populations. The social, economic and political contexts in which implementation research is conducted will continue to determine the focus of research, disbursement of research funding or oppression of marginalized and key populations [4, 12, 13, 14, 15]. Third, the influence of political and corporate interests has also eroded public trust in scientific knowledge [16].

Citizen science has historically been associated with involving the public in collecting or analysing large amounts of data, with a goal to educate citizens through their participation [17]. Citizen science is often described as a specific methodology that falls under the umbrella of community‐based participatory research approaches, which centres community involvement throughout the research process. One aspect that sets citizen science apart from other methods is its focus on engagement with individual community members outside of formal community‐academic partnerships [18]. This is especially important given how intracommunity dynamics of stigmatization or other barriers to participation may lead to only certain segments of communities being represented by such partnerships [19].

Citizen science can address the above‐mentioned limitations faced in implementation science in several ways. First, citizen‐led approaches promote a democratization of the research process. This enables research to be informed by the lived experiences of citizens which subsequently enhances public trust in scientific research [20, 21]. Citizen‐led and community‐involved approaches can harness community resources and empower community members, to advance implementation science research in support of marginalized or key populations [22]. Second, citizen science approaches enable research efforts to be executed and sustained with minimal funding [23]. The need for fewer resources will allow implementation science studies to explore areas that may not traditionally attract funding, such as those concerning marginalized sub‐populations like key populations affected by HIV. Finally, besides being potentially cost‐effective, this community involvement also has the potential to build scientific literacy among lay people and improve their knowledge of the subject matter [24]. In this commentary, we discuss how citizen science approaches can be leveraged to spur equity in HIV implementation science. Specifically, we sought to briefly conceptualize and define citizen science, propose a framework for situating a citizen‐engaged HIV implementation science and provide some examples of how this can be achieved.

## 2 DISCUSSION

### 2.1 Conceptualizing citizen science

Although citizen science is premised on the active involvement of citizens in scientific research, the definition and conceptualization of citizen science has been highly varied and is dependent on context [25]. Scholars have also pointed out that a narrow definition of citizen science is antithetical to the innovative and bottom‐up processes that generate knowledge in citizen science [26]. It is in this spirit that we adopt a broad definition of citizen science in this commentary and define it as the involvement of non‐academic or medical researchers in scientific processes.

Despite advocating for a broad, dynamic definition of citizen science, scholars have, in the past few decades, attempted to develop principles that align with the practice of citizen science. These evidence‐based typologies and frameworks determine were developed through participatory processes, and delineate how best communities can be meaningfully involved in implementation studies [27]. For example, scholars in the United States have developed eight stakeholder engagement principles that can be used to determine the extent to which research is community‐engaged [28]. A more global effort saw the development of 10 principles of citizen science that were developed by an international community of citizen science practitioners and researchers, which highlight the roles that citizens play, expected outcomes, benefits, limitations and biases, data sharing agreements, and levels of participation and acknowledgement that citizens should have in a scientific process [27].

In practice, how have citizens typically been involved in scientific processes? Citizen involvement varies from utilizing volunteers for data collection and consulting community members when designing research studies to empowering community members to shape research questions and methodologies [25, 29, 30]. Several participatory tools such as co‐creation or co‐production groups [31] and crowdsourcing approaches have been utilized to engage citizens and communities in several aspects of solving health‐related problems and developing solutions to them [32]. Citizens have also been involved in the implementation of interventions. These include involving communities in the planning, developing, delivering, evaluating, and scaling up of clinical HIV and sexually transmitted infections services [33], as well as leveraging individual (e.g. individual community member involvement in research activities), interpersonal (e.g. social capital, social networks, peer networks) and organization, community, and population (e.g. multisectoral, multifacility collaborations, community dialogues) level dynamics and resources [34]. Nevertheless, scholars have more recently highlighted that in many cases, implementation studies that engage the community only do so in the later stages of research, and not in postulating research questions or hypotheses [28].

We define the concept of a citizen‐engaged HIV implementation science as one that involves citizens and communities deeply in HIV implementation science activities. To illustrate this, we describe how participatory and citizen scientific approaches and tools have been adopted in several areas of implementation science. We draw on three areas previously defined by Geng and colleagues that serve to drive impactful implementation science in the HIV response to frame this section; first, “whose perspectives?”, “what questions are being asked?” and “how are questions asked?” [35].

### 2.2 Whose perspectives? Citizen engagement in question‐generating processes

In choosing to engage citizens and communities throughout the research process, researchers centre the needs and perspectives of the most relevant stakeholders in every stage of research design and execution, and this is particularly true in the question‐generation process. Several approaches have been undertaken to ensure that citizens and communities are deeply engaged in participatory processes. We posit that such approaches involving diverse partners, typically including key populations affected by HIV, policymakers, healthcare providers and researchers, ensure that end‐user voices are incorporated into the process of formulating of scientific questions, and therefore, the flow of funding into areas of research that are prioritized by communities.

Co‐creation or co‐production groups have been used as a method to integrate citizens and communities into the research question generation, research design and process. Co‐creation processes typically involve equal collaboration between researchers and citizens or community members and are characterized by joint decision‐making on the goals, processes and outcomes of research [31]. Increasingly, crowdsourcing has been adopted as a co‐creation option to integrate community's opinions while building capacity among community members to advance their community's needs through academic inquiries. Crowdsourcing is a community‐based participatory activity that has the public develop and solve problems, facilitating the implementation of the solution in the process. Crowdsourcing comprises activities such as the use of open calls or challenge contests, or designathons (intensive, structured and collaborative activities to develop solutions in a short period of time) [36]. A systematic review of crowdsourcing for health and medical research has found that such approaches have been effective at improving behavioural outcomes in health, and that there is some evidence that such approaches may be more cost‐effective than conventional expert‐driven approaches [32].

For example, in a study conducted by Sha and colleagues [37], the team adopted a crowdsourcing competition where they launched an open call for concepts and ideas to develop intervention packages to facilitate greater HIV pre‐exposure prophylaxis (PrEP) adherence in China. While crowdsourcing activities traditionally define a general scope of the problem to be tackled, participants are invited to further refine the scope of the problem into specific areas they wish to address and provide solutions accordingly. Crowdsourcing procedures are selected to address public health problems because they facilitate community buy‐in to final solutions. The buy‐in occurs with the active involvement of the community, including key populations affected by HIV, in the submission of ideas, developing tailored interventions and engagement in the implementation of these interventions. Further, in adopting these approaches, stakeholders are able to enhance capacity, education and mentorship, and identify talented trainees among community members involved in the co‐creation process. In doing so, these approaches help develop trained personnel within the community to continue the relevant research inquiries even after the crowdsourcing project, sustainably advancing the needs and welfare of the relevant community [38].

### 2.3 What questions are being asked? Citizen‐led research on implementation strategies

The process of ensuring that evidence‐based practices are translated and implemented effectively into routine health practices involves research that goes beyond effectiveness studies and onto implementation strategies themselves. These also involve research on the de‐implementation of harmful or low‐value practices, as well as about contexts to improve the transferability of implementation strategies. Several efforts have been made to incorporate citizen science approaches to pursuing implementation research.

Citizen science research approaches have been used to address or overcome limitations in existing implementation science evaluative frameworks. For example, the Reach and Effectiveness, Adoption and Implementation, and Maintenance (RE‐AIM) model aims to address research‐to‐practice gaps in public health settings where individual‐level and staff/setting‐level considerations are accounted for when evaluating evidence‐based interventions [39]. Shelton and colleagues have argued that the existing conceptualization of RE‐AIM adopts a static perspective of sustained impact, failing to account for diverse populations where a single implementation strategy may be implemented, as well as dynamic changes that influence the actual sustainability of an implementation strategy [40]. Instead, that RE‐AIM framework needs to be adapted to include considerations of fidelity consistency across diverse populations, to document and address inequities as they arise in any or all domains of RE‐AIM dimensions, and to extend the notion of “maintenance” to conceptualizations of longer‐term intervention sustainability to address dynamic (i.e. changing) contextual factors and examine multi‐level determinants of sustainability.

Another popular implementation science framework is the Consolidated Framework for Implementation Research (CFIR). As a “determinants framework,” CFIR demands researchers to consider intervention characteristics (what does the intervention entail), outer setting (what are external contexts influencing the implementation process), inner setting (what are internal contexts influencing the implementation process), characteristics of individuals (actions and behaviours of involved stakeholders) as well as the process of implementation (implementation strategies) [41, 42, 43].

Researchers have begun considering how citizen science‐driven approaches may address gaps in such implementation science frameworks. Kaul and colleagues have published an implementation science protocol that aims to adopt an equity and sustainability‐informed lens throughout the use of both the RE‐AIM and CFIR frameworks. By using an equity‐based implementation science approach, diverse avenues of communication and collaborations with community groups drove the design of the EquiPrEP programme at New York, which aims to optimize the use of long‐acting injectable HIV pre‐exposure prophylaxis [44].

### 2.4 How are questions asked? Citizen science as a context‐dynamic process

Finally, ensuring that HIV implementation research provides scientific perspectives that facilitate the sharing of insight across contexts and settings is important to spur the implementation of evidence‐based practices. Citizen science as an approach, as described earlier, shares a similar issue where a balance must be struck between unique contexts valued for their unique and organic processes and contributions to knowledge production, and generating transferable and insightful knowledge that can apply to multiple settings.

Guidelines and principles help facilitate the generation of transferable and insightful knowledge across diverse contexts without being overly prescriptive. Such guidelines should recognize unique challenges from diverse communities and provide guidance that can be tailored to unique contexts. For example, UNAIDS provides technical guidance on setting up community‐led monitoring (CLM) of HIV services through a 5‐stage model that expands on key processes within the monitoring process [45]. Beyond drawing insights from existing CLM initiatives and key informant inputs in establishing these guidelines, the guidelines highlight the importance of flexibility towards the specific context of resources and capacities. These recognize the diverse challenges that various contexts may face (e.g. societal and structural barriers). Other programmes that highlight the use of guideline‐based implementation research include the PRECluDE consortium on chronic disease co‐morbidities in HIV populations (2020), as well as the LINKAGES programme [46], where guidelines covering important facets (e.g. population engagement and empowerment, peer outreach) have been set up for implementation programmes for HIV key populations. Taken together, the use of guideline and principles can help in setting up a framework for which transferable knowledge and insights can be generated across diverse contexts—important given the context‐dynamic features of engaging in citizen science approaches.

Moreover, complex social, cultural and psychological factors influence HIV implementation research. Drawing from dynamic and flexible methodologies across diverse disciplines such as social sciences and humanities in setting up citizen‐led HIV implementation research can aid in the development of a more comprehensive understanding and production of more equitable outcomes across various contexts. The employment of participatory approaches in research recognizes the value that citizen scientists provide, which includes context‐specific knowledge that can guide the research process. For example, Dilley and colleagues [47] were able to tap into participatory approaches in data collection and monitoring, which allowed for more effective and accurate collection of physical activity patterns within the community, enhancing the likely effectiveness of community‐level intervention. Participatory approaches can also be adopted at the implementation level, such as in community‐based intervention programmes through task‐shifting and capacity building [48]. Additionally, the consideration of methodological concepts across diverse disciplines can aid in the research process. This can include recognizing concepts of power, process and relationships within participatory processes [49], which can affect the quality of data as well as research outcomes.

## 3 CONCLUSIONS

Table 1 summarizes an expanded framework for how citizen‐engaged approaches can strengthen HIV implementation science, based on our discussion above. It is worth noting that there are several challenges, including stereotypes and mistrust between academic and community partners, the lack of alignment between funder priorities and participatory approaches, and structural issues such as unbalanced knowledge hierarchies and data rights [50, 51, 52]. These challenges notwithstanding, citizen‐engaged approaches offer substantial benefits in bridging inequities in our HIV response. By involving communities and citizens in the development and growth of HIV implementation science, we can ensure that our implementation approaches remain equitable and committed to bridging divides and ending AIDS as a public health threat.

**Table 1:** Situating a citizen‐engaged HIV implementation science

Areas of focus	HIV implementation science	Citizen‐engaged HIV implementation science	Benefits
**Whose perspectives?**	Ensuring that end‐users are represented in question‐generating processes. Needs of relevant stakeholders are centred at every stage of the implementation research process.	Using participatory tools, such as co‐creation, co‐production and crowdsourcing approaches, to engage the public and key populations affected by HIV in identifying problems, solve health problems and implement solutions.	Questions for implementation science are not only guided by experts, but a diverse pool of citizens in the community and among key populations affected by HIV.
**What questions are being asked?**	Going beyond research on effectiveness of interventions, towards research on implementation strategies, de‐implementation of harmful or low‐value strategies and implementation contexts.	Synthesizing citizen or community‐led approaches with existing implementation science frameworks and approaches (e.g. CFIR, RE‐AIM); ensuring communities and key populations affected by HIV have a say in interrogating and deconstructing such frameworks and adapting them to local contexts.	Ensures that our theories, models and frameworks in implementation science reflect the needs of end‐users and especially key populations affected by HIV.
**How are questions asked?**	Despite unique implementation contexts, ensuring that research insights are standardized and offer lessons learnt that can be generalized and applied across contexts.	Adopting broad, guiding principles and frameworks that account for dynamic contexts to promote citizen‐engaged research in HIV implementation science. Avoiding narrow definitions that limit the creativity, innovation and “ground‐up” wisdom of local citizens and key populations affected by HIV.	Encourages best practice‐sharing in citizen‐engaged approaches to HIV implementation science across contexts.

Abbreviations: CFIR, Consolidated Framework for Implementation Research model; RE‐AIM, Reach and Effectiveness, Adoption and Implementation, and Maintenance model.

## COMPETING INTERESTS

The authors declare no competing interests.

## AUTHORS’ CONTRIBUTIONS

SYC, DWSH, YXW, MHJ and RKJT conceptualized the commentary; BXHT, SYC, DWSH, YXW and RKJT wrote the first draft of the manuscript. All authors have read and approved the final manuscript.

## CME STATEMENT

This article is published as part of a supplement supported by unrestricted educational grant by ViiV Healthcare.

Credits Available for this Activity: American Medical Association (AMA Credit).

Washington University School of Medicine in St. Louis designates this enduring material for a maximum of 1 AMA PRA Category 1 Credit™. Physicians should claim only the credit commensurate with the extent of their participation in the activity.

## Data Availability

Data sharing is not applicable as no new data are generated, or the article describes entirely theoretical research.

## References

[1] Bauer MS , Kirchner J . Implementation science: what is it and why should I care? Psychiatry Res. 2020;283:112376. 31036287 10.1016/j.psychres.2019.04.025

[2] Kerkhoff AD , Muiruri C , Geng EH , Hickey MD . A world of choices: preference elicitation methods for improving the delivery and uptake of HIV prevention and treatment. Curr Opin HIV AIDS. 2023;18(1):32–45. 36409315 10.1097/COH.0000000000000776 PMC9772083

[3] Eccles MP , Mittman BS . Welcome to implementation science. Implement Sci. 2006;1(1).

[4] Brownson RC , Shelton RC , Geng EH , Glasgow RE . Revisiting concepts of evidence in implementation science. Implement Sci. 2022;17(1):26. 35413917 10.1186/s13012-022-01201-y PMC9004065

[5] Chang LW , Serwadda D , Quinn TC , Wawer MJ , Gray RH , Reynolds SJ . Combination implementation for HIV prevention: moving from clinical trial evidence to population‐level effects. Lancet Infect Dis. 2013;13(1):65–76. 23257232 10.1016/S1473-3099(12)70273-6 PMC3792852

[6] World Health Organisation . Prevention and treatment of HIV and other sexually transmitted infections among men who have sex with men and transgender people: recommendations for a public health approach. 2011. 26158187

[7] Beyrer C , Baral S , Kerrigan D , El‐Bassel N , Bekker LG , Celentano DD . Expanding the space: inclusion of most‐at‐risk populations in HIV prevention, treatment, and care services. J Acquir Immune Defic Syndr. 2011;57(Suppl 2):S96–S99. 21857306 10.1097/QAI.0b013e31821db944 PMC3164959

[8] Fay H , Baral SD , Trapence G , Motimedi F , Umar E , Iipinge S , et al. Stigma, health care access, and HIV knowledge among men who have sex with men in Malawi, Namibia, and Botswana. AIDS Behav. 2011;15(6):1088–1097. 21153432 10.1007/s10461-010-9861-2

[9] Glasgow RE , Eckstein ET , Elzarrad MK . Implementation science perspectives and opportunities for HIV/AIDS research: integrating science, practice, and policy. J Acquir Immune Defic Syndr. 2013;63(Suppl 1):S26–S31. 23673882 10.1097/QAI.0b013e3182920286

[10] Mody A , Sohn AH , Iwuji C , Tan RKJ , Venter F , Geng EH . HIV epidemiology, prevention, treatment, and implementation strategies for public health. Lancet. 2024;403(10425):471‐492. 38043552 10.1016/S0140-6736(23)01381-8

[11] Huebschmann AG , Leavitt IM , Glasgow RE . Making health research matter: a call to increase attention to external validity. Annu Rev Public Health. 2019;40:45–63. 30664836 10.1146/annurev-publhealth-040218-043945

[12] Montini T , Graham ID . “Entrenched practices and other biases”: unpacking the historical, economic, professional, and social resistance to de‐implementation. Implement Sci. 2015;10:24. 25889285 10.1186/s13012-015-0211-7 PMC4339245

[13] Kempner J , Perlis CS , Merz JF . Forbidden knowledge. Science. 2005;307(5711):854. 15705829 10.1126/science.1107576

[14] Frickel S , Gibbon S , Howard J , Kempner J , Ottinger G , Hess DJ . Undone science: charting social movement and civil society challenges to research agenda setting. Sci Technol Hum Values. 2010;35(4):444–473. 10.1177/0162243909345836 PMC7041968 32099268

[15] Woodhouse E , Hess D , Breyman S , Martin B . Science studies and activism: possibilities and problems for reconstructivist agendas. Soc Stud Sci. 2002;32(2):297–319.

[16] Bäckstrand K . Civic science for sustainability: reframing the role of experts, policy‐makers and citizens in environmental governance. Glob Environ Polit. 2003;3(4):24–41.

[17] Rosas LG , Espinosa PR , Jimenez FM , King AC . The role of citizen science in promoting health equity. Annu Rev Public Health. 2022;43(1):215–234. 34724389 10.1146/annurev-publhealth-090419-102856 PMC9034747

[18] Toubiana M , Ruebottom T . Stigma hierarchies: the internal dynamics of stigmatization in the sex work occupation. Adm Sci Q. 2022;67(2):515–552.

[19] Amirrudin A , Harrigan N , Naqvi I . Scaled, citizen‐led, and public qualitative research: a framework for citizen social science. Curr Sociol. 2023;71(6):1019–1039.

[20] Strasser B , Haklay M . Citizen science: expertise, democracy, and public participation. 2018.

[21] Moore GF , Audrey S , Barker M , Bond L , Bonell C , Hardeman W , et al. Process evaluation of complex interventions: Medical Research Council guidance. Br Med J. 2015;350:h1258. 25791983 10.1136/bmj.h1258 PMC4366184

[22] Silvertown J . A new dawn for citizen science. Trends Ecol Evol. 2009;24(9):467–471. 19586682 10.1016/j.tree.2009.03.017

[23] Bonney R , Phillips TB , Ballard HL , Enck JW . Can citizen science enhance public understanding of science? Public Underst Sci. 2016;25(1):2–16. 26445860 10.1177/0963662515607406

[24] Nascimento S , Rubio Iglesias J , Owen R , Schade S , Shanley L . Citizen science for policy formulation and implementation. UCL Press; 2018.

[25] Haklay MM , Dörler D , Heigl F , Manzoni M , Hecker S , Vohland K . What is citizen science? The challenges of definition. In The science of citizen science. 2021.

[26] Auerbach J , Barthelmess EL , Cavalier D , Cooper CB , Fenyk H , Haklay M , et al. The problem with delineating narrow criteria for citizen science. Proc Natl Acad Sci USA. 2019;116(31):15336–15337. 31363074 10.1073/pnas.1909278116 PMC6681715

[27] Robinson LD , Cawthray JL , West SE , Bonn A , Ansine J . Ten principles of citizen science. Citizen science: innovation in open science, society and policy. UCL Press; 2018.

[28] Goodman MS , Ackermann N , Bowen DJ , Thompson VS . Reaching consensus on principles of stakeholder engagement in research. Prog Community Health Partnersh. 2020;14(1):117. 32280129 10.1353/cpr.2020.0014 PMC7867997

[29] Sutcliffe H . A report on responsible research and innovation. MATTER and the European Commission; 2011.

[30] Israel BA , Parker EA , Rowe Z , Salvatore A , Minkler M , López J , et al. Community‐based participatory research: lessons learned from the Centers for Children's Environmental Health and Disease Prevention Research. Environ Health Perspect. 2005;113(10):1463–1471. 16203263 10.1289/ehp.7675 PMC1281296

[31] Stark E , Ali D , Ayre A , Schneider N , Parveen S , Marais K , et al. Coproduction with autistic adults: reflections from the authentistic research collective. Autism Adulthood. 2020;3(2):195–203. 10.1089/aut.2020.0050 PMC8992895 36601467

[32] Wang C , Han L , Stein G , Day S , Bien‐Gund C , Mathews A , et al. Crowdsourcing in health and medical research: a systematic review. Infect Dis Poverty. 2020;9(1):8. 31959234 10.1186/s40249-020-0622-9 PMC6971908

[33] Tan RKJ , Marley G , Kpokiri EE , Wang T , Tang W , Tucker JD. Participatory approaches to delivering clinical sexually transmitted infections services: a narrative review. Sex Health. 2022;19:299‐308. 35942569 10.1071/SH22039

[34] Li C , Zhao P , Tan RKJ , Wu D . Community engagement tools in HIV/STI prevention research. Curr Opin Infect Dis. 2024;37(1):53–62. 38050762 10.1097/QCO.0000000000000993

[35] Geng EH , Nash D , Phanuphak N , Green K , Solomon S , Grimsrud A , et al. The question of the question: impactful implementation science to address the HIV epidemic. J Int AIDS Soc. 2022;25(4):e25898. 35384312 10.1002/jia2.25898 PMC8982316

[36] WHO/TDR . Crowdsourcing in health and health research: a practical guide. Switzerland: World Health Organisation; 2018.

[37] Sha Y , Li C , Xiong Y , Hazra A , Lio J , Jiang I , et al. Co‐creation using crowdsourcing to promote PrEP adherence in China: study protocol for a stepped‐wedge randomized controlled trial. BMC Public Health. 2022;22(1):1697. 36071401 10.1186/s12889-022-14117-5 PMC9449927

[38] Tahlil KM , Obiezu‐Umeh C , Gbajabiamila T , Nwaozuru U , Oladele D , Musa AZ , et al. A designathon to co‐create community‐driven HIV self‐testing services for Nigerian youth: findings from a participatory event. BMC Infect Dis. 2021;21(1):505. 34059014 10.1186/s12879-021-06212-6 PMC8166032

[39] Glasgow RE , Vogt TM , Boles SM . Evaluating the public health impact of health promotion interventions: the RE‐AIM framework. Am J Public Health. 1999;89(9):1322–1327. 10474547 10.2105/ajph.89.9.1322 PMC1508772

[40] Shelton RC , Chambers DA , Glasgow RE . An extension of RE‐AIM to enhance sustainability: addressing dynamic context and promoting health equity over time. Front Public Health. 2020;8:134. 32478025 10.3389/fpubh.2020.00134 PMC7235159

[41] Damschroder LJ , Aron DC , Keith RE , Kirsh SR , Alexander JA , Lowery JC . Fostering implementation of health services research findings into practice: a consolidated framework for advancing implementation science. Implement Sci. 2009;4(1):50. 19664226 10.1186/1748-5908-4-50 PMC2736161

[42] Smit DJM , Proper KI , Engels JA , Campmans JMD , van Oostrom SH . Barriers and facilitators for participation in workplace health promotion programs: results from peer‐to‐peer interviews among employees. Int Arch Occup Environ Health. 2023;96(3):389–400. 36305914 10.1007/s00420-022-01930-z PMC9614189

[43] Grootjans SJM , Stijnen MMN , Hesdahl DEJI , Kroese M , Ruwaard D , Jansen MWJ . Implementation of an integrated community approach in deprived neighbourhoods: a theory‐based process evaluation using the Consolidated Framework for Implementation Research (CFIR). Scand J Public Health. 2023. 10.1177/14034948231199804 PMC11481404 37726916

[44] Kaul CM , Moore BE , Kaplan‐Lewis E , Casey E , Pitts RA , Pagan Pirallo P , et al. EquiPrEP: an implementation science protocol for promoting equitable access and uptake of long‐acting injectable HIV pre‐exposure prophylaxis (LAI‐PrEP). PLoS One. 2023;18(9):e0291657.EquiPrEP: an implementation science protocol for promoting equitable access and uptake of long‐acting injectable HIV pre‐exposure prophylaxis (LAI‐PrEP) 37725628 10.1371/journal.pone.0291657 PMC10508596

[45] UNAIDS . Establishing community‐led monitoring of HIV services. 2021.

[46] Family Health International 360 . Key Population Program Implementation Guide. 2017.

[47] Dilley JR , Moore JB , Summers P , Price AA , Burczyk M , Byrd L , et al. A citizen science approach to determine physical activity patterns and demographics of greenway users in Winston-Salem, North Carolina. International journal of environmental research and public health, 2019;16(17):3150. 31470544 10.3390/ijerph16173150 PMC6747415

[48] Okop K , Delobelle P , Lambert EV , Getachew H , Howe R , Kedir K , et al. Implementing and evaluating community health worker‐led cardiovascular disease risk screening intervention in sub‐Saharan Africa communities: a participatory implementation research protocol. Int J Environ Res Public Health. 2022;20(1). 10.3390/ijerph20010298 PMC9819933 36612620

[49] Chung K , Lounsbury DW . The role of power, process, and relationships in participatory research for statewide HIV/AIDS programming. Soc Sci Med. 2006;63(8):2129–2140. 16828213 10.1016/j.socscimed.2006.04.035

[50] Freeman ER , Brugge D , Bennett‐Bradley WM , Levy JI , Carrasco ER . Challenges of conducting community‐based participatory research in Boston's neighborhoods to reduce disparities in asthma. J Urban Health. 2006;83(6):1013–1021. 17103339 10.1007/s11524-006-9111-0 PMC3261288

[51] Benyei P , Skarlatidou A , Argyriou D , Hall R , Theilade I , Turreira‐García N , et al. Challenges, strategies, and impacts of doing citizen science with marginalised and indigenous communities: reflections from project coordinators. In Citizen science: theory and practice. 2023.

[52] Lewis D . Barriers to citizen science and dissemination of knowledge in healthcare. In Citizen science: theory and practice. 2022.

